# A framework for generalized subspace pattern mining in high-dimensional datasets

**DOI:** 10.1186/s12859-014-0355-5

**Published:** 2014-11-21

**Authors:** Edward WJ Curry

**Affiliations:** Division of Cancer, Imperial College London, Hammersmith Hospital, Du Cane Road, London, W12 0NN UK

## Abstract

**Background:**

A generalized notion of biclustering involves the identification of patterns across subspaces within a data matrix. This approach is particularly well-suited to analysis of heterogeneous molecular biology datasets, such as those collected from populations of cancer patients. Different definitions of biclusters will offer different opportunities to discover information from datasets, making it pertinent to tailor the desired patterns to the intended application. This paper introduces ‘GABi’, a customizable framework for subspace pattern mining suited to large heterogeneous datasets. Most existing biclustering algorithms discover biclusters of only a few distinct structures. However, by enabling definition of arbitrary bicluster models, the GABi framework enables the application of biclustering to tasks for which no existing algorithm could be used.

**Results:**

First, a series of artificial datasets were constructed to represent three clearly distinct scenarios for applying biclustering. With a bicluster model created for each distinct scenario, GABi is shown to recover the correct solutions more effectively than a panel of alternative approaches, where the bicluster model may not reflect the structure of the desired solution. Secondly, the GABi framework is used to integrate clinical outcome data with an ovarian cancer DNA methylation dataset, leading to the discovery that widespread dysregulation of DNA methylation associates with poor patient prognosis, a result that has not previously been reported. This illustrates a further benefit of the flexible bicluster definition of GABi, which is that it enables incorporation of multiple sources of data, with each data source treated in a specific manner, leading to a means of intelligent integrated subspace pattern mining across multiple datasets.

**Conclusions:**

The GABi framework enables discovery of biologically relevant patterns of any specified structure from large collections of genomic data. An R implementation of the GABi framework is available through CRAN (http://cran.r-project.org/web/packages/GABi/index.html).

**Electronic supplementary material:**

The online version of this article (doi:10.1186/s12859-014-0355-5) contains supplementary material, which is available to authorized users.

## Background

Widespread adoption of genome-wide profiling technologies in biological research has led to a proliferation of high dimensional datasets, involving simultaneous collection of tens of thousands of molecular measurements from individual biological samples. Unsupervised clustering has proved to be a successful approach to the analysis of such data, as typified by gene expression microarrays, with widespread use following an initial application in [1]. But where cluster analysis identifies groups of elements with related values across a dataset, *biclustering* refers to a formalisation of the approach of searching for groups of elements with values exhibiting similar behaviour across some subset of a dataset.

Heterogeneity in biological systems, where different cells (or groups of cells) elicit different mechanisms to achieve a shared behaviour, is becoming more apparent as more molecular profiling is performed. This is perhaps most noted in the field of cancer research, where many cancers have been shown to separate into largely distinct molecular subtypes [2-5]. Furthermore, tumours of a single molecular subtype defined by hierarchical clustering of gene expression profiles can still exhibit a considerable degree of heterogeneity at levels of genetic sequence and copy number, epigenetic modifications, gene and protein expression [6]. A recent illustrative example from breast cancer [3] demonstrates 10 molecular subtypes discovered through clustering of profiles from 2,000 tumour samples, which do not clearly fit with 4 previously described molecular subtypes that were discovered through cluster-based analysis of gene expression profiles [2]. The ability to discover relationships that may not be evident across the full set of samples in a dataset makes *biclustering* methods particularly well-suited to the analysis of large heterogeneous biological datasets. This is particularly relevant in cancer research, which typically involve high levels of molecular and genetic heterogeneity, as is demonstrated in a recent application of biclustering [7]. With the large amounts of such data avaliable there is tremendous potential for discovering patterns that inform us of some key clinical or biological feature. But to achieve this, it would help greatly to be able to discover *any* desired pattern across subsets of large data collections.

Using the framework of [8], we define the biclustering problem as follows:

(1) $$\begin{array}{@{}rcl@{}}{} \text{Say we have data matrix}\; A&& \end{array} $$

(2) $$\begin{array}{@{}rcl@{}} \text{with rows}\; X &=& \{ x_{1},...,x_{n}\}, \end{array} $$

(3) $$\begin{array}{@{}rcl@{}} \text{and columns}\; Y &=& \{ y_{1},...,y_{m}\} \end{array} $$

(4) $$\begin{array}{@{}rcl@{}} \text{Define a bicluster}\; (I,J)&& \end{array} $$

(5) $$\begin{array}{@{}rcl@{}} \text{where}\; I &=& \{ i_{1},...,i_{p}\}, I \subseteq X, p\leq n, \end{array} $$

(6) $$\begin{array}{@{}rcl@{}} \text{and}\; J &=& \{ j_{1},...,j_{q}\}, J \subseteq Y, q\leq m. \end{array} $$

(7) $$\begin{array}{@{}rcl@{}} \text{We denote}\; A_{IJ} &=& \left[ a_{ij}\right].\forall i \in I, j \in J. \end{array} $$

Then, for some evaluation of desirability of biclusters, say *f*(*I*,*J*), the goal is to find the optimal ordering of all possible biclusters, say *Ψ*^′^. The total number of possible biclusters can be vast for a large dataset, as each ordering *Ψ*∈*℘*(*X*)×*℘*(*Y*). In fact, the problem of finding a set of biclusters (whether they are exclusive or overlapping) to cover a data matrix is known to be NP-hard [8], as it is a generalisation of the problem of finding a minimum set of bicliques to cover a bipartite graph.

Biclustering was introduced in the 1970’s [9], but received relatively little attention until Cheng & Church applied it to the analysis of gene expression data (in [10]), using the term ‘biclustering’ to describe the approach. Since then there have been a large number of methods developed for finding biclusters in data matrices, each with its own data model and method of optimizing that model, including [11-18]. Surveys of biclustering algorithms can be found in [8,19,20], including some illuminating comparisons based on datasets constructed to provide benchmarks. While most biclustering algorithms focus on an individual data model, there could be a huge range of different uses of bicluster analysis: any situation in which evaluating some localised pattern within a heterogeneous dataset could be of interest. However, much of the literature on biclustering refers to 4 distinct types of bicluster, as defined in [8].

1. Constant: all values are (close to) the same
2. Constant rows/columns: all values across each row (or across each column) are close to the same
3. Coherent values: a single shifting/scaling pattern is preserved across all rows
4. Evolutive: the same sequence of ‘up’ and ‘down’ trends is preserved across each row and/or column.

The coherent biclusters are sometimes split into ‘additive’ biclusters (shifting patterns) and ‘multiplicative’ biclusters (scaling patterns). These bicluster models (and the examples presented in each of [8,19,20]) can be summarized into two general scenarios: (i) those in which the values for each row of the bicluster, that is *A*_*iJ*_, are closer to each other than to the values of row *i* across the rest of the columns (∀*y*∉*J*); (ii) those in which the values for each row of the bicluster follow the same trend across columns *J*, ∀*i*∈*I*. According to the bicluster types given in the previous paragraph, types 1 & 2 represent scenario (i), types 3 & 4 represent scenario (ii). The ‘plaid’ model of [11] involves offsets for both rows and columns, and the whole bicluster, and therefore contains features of both scenarios. HSSVD [21] is an interesting recent approach in that it encodes two different structures of bicluster that it identifies simultaneously: ‘mean-biclusters’, which correspond to bicluster types 1 & 2, and ‘variance-biclusters’, which may represent type 3 but also applies to biclusters that don’t fit any of the types 1-4.

As large number of algorithms exist for identifying biclusters in data matrices, a selection of the most popular are outlined here. The focus of this summary is the model defining the type(s) of bicluster discovered by each algorithm, as it is the current limitation of the state-of-the-art in this area that the GABI framework was designed to address.

*Cheng & Church:* [10] A bicluster model is defined using a score termed ‘mean squared residue’, which is the sum of the squared differences of each element from its row-average and column-average across the bicluster. A specified maximum allowed mean squared residue score is set, and biclusters are found through a greedy process by iteratively removing the rows and columns from the entire data matrix with the highest mean squared residue values. This approach can therefore discover biclusters of type 1, 2 and 3, but not 4.

*Plaid:* [11] A generative model for a whole (numeric) data matrix is formulated and fitted through iterative optimization of the parameters so as to minimize error. There is a whole-dataset offset (*θ*) for all elements, a per-bicluster offset (*μ*_*k*_) for all elements within bicluster *k*, per-row offsets (*α*_*ik*_) for all elements of row *i* in bicluster *k*, per-column offsets (*β*_*jk*_) for all elements of row *j* in bicluster *k*, and random noise for each element. The flexibility of this generative model allows the Plaid method to discover biclusters of type 1, 2 and 3.

*Bimax:* [19] The data matrix is ‘binarized’ so that each value is 1 or 0, and the bicluster model is defined as any submatrix consisting entirely of 1s. The binarized data matrix is divided recursively until all biclusters are identified. Performance of the Bimax algorithm on any real task is dependent on the binarization approach, and its application is restricted to situations where the patterns of interest can be represented in a binary form, which is a limited case of type 1 as defined above.

*ISA:* [15] Input data is normalized so that the mean of each row (across the full set of columns) is zero. Then a ‘module’ (i.e. a bicluster) is {*A*,*B*} such that ∀*a*∈*A*, $\displaystyle \frac {1}{|B|}\sum \limits _{b \in B}X_{\textit {ab}}$ is greater than some threshold. The algorithm proceeds by initializing a random subset *A*_0_, then identifying *B*_0_ as those columns for which the average value across *A*_0_ is greater than the threshold. With *B*_0_ defined, *A*_1_ is identified as those rows for which the above condition is satisfied. *B*_1_ is identified using *A*_1_, and the process continues until *A*_*n*+1_=*A*_*n*_=*A*^′^. This process is repeated for a large number of random initializations $A_{0}^{*}$, after which any *A*∗^′^ that are highly overlapping are merged together. The module definition means that ISA discovers only biclusters of type 1 or type 2 (constant rows only). Interestingly, ISA is shown to be related to SVD, a modification of which was used as the basis of HSSVD.

It was noted in [19] that a fair comparison of biclustering methods is inherently difficult, owing to the fact that each is designed for a particular situation, and thus it would be rare to find any cases that truly represent a ‘like-with-like’ comparison. If one’s goal is to produce a biclustering method that can discover biclusters of a type that fits into either of these scenarios, then comparisons using the benchmark datasets of [19,20] would be an important part of demonstrating the contribution of the new method to the literature. However, owing to the diverse range of possible applications of biclustering, it would be expected that there exist applications for which one would wish to adopt a bicluster model that is specifically intended for the particular inferences desired to be made on the basis of the output. Therefore, the goal of this current paper is to introduce an implementation of a biclustering framework that is so general that it could be adapted to any possible situation, including many which do not fit into the two scenarios outlined in the preceding paragraph. Steps toward this goal were made in a recent evolutionary biclustering approach, termed Evo-Bexpa [22], which allows the user to specify preferences on a number of properties of the biclusters to be discovered. However, this flexibility is still limited to only four characteristics (degree of correlation between rows, product of the number of rows and columns, degree of overlap between biclusters, and variance across rows within a bicluster) and therefore does not represent a truly customizable biclustering algorithm for generalized subspace pattern mining. It is the absence of a suitable tool for completely adaptable subspace pattern mining that I address in this paper.

A heuristic approach to solving a problem involves adopting methods that aren’t guaranteed to find the best possible solution or aren’t guaranteed to find a solution within a desirable limit of computation time, but find a ‘good’ solution within ‘reasonable’ time in the vast majority of cases that are confronted. Genetic algorithms [23] are an example of a ‘global search heuristic’ that involves forming a pool of whole solutions at each step and improving these by various methods, which avoid the tendency to converge on locally optimal solutions that are far from the best overall solution [24]. GAs have been applied to the biclustering problem in [25], but the high dimensionality of molecular and genetic profiling datasets result in most of the effort in exploring the search space going on variable selection. An important benefit of the GA framework is that the bicluster definition and evaluation procedures are explicitly and modularly incorporated into the search algorithm, enabling flexible specification of the bicluster model. The framework can therefore be customized for application to different data mining tasks, and can be used for the direct comparison of different bicluster models.

Ovarian cancer is associated with a poor prognosis and only around 40% of women diagnosed with ovarian cancer are alive after 5 years [26]. Conventional first-line treatment involves cytoreductive surgery and chemotherapy with platinum-based compounds. Initial response rates are good and some patients can continue to respond to multiple rounds of treatment, but in most cases the disease eventually recurs in a chemo-resistant form [26,27]. It has been shown that cancer cells tend to lose coherent regulation of DNA methylation across regions of the genome, resulting in widespread variation in the levels of DNA methylation at particular sites and stochastic patterns of gene expression [28]. In fact, identification of dysregulation of DNA methylation in cancer was the subject of a recent exciting application of biclustering [21]. However, it has not previously been investigated whether or not, within a set of cancer patients, widespread dysregulation of DNA methylation (i.e. a lack of *concordance* in methylation levels) has an impact on patient outcome. The GABi framework is used here to demonstrate that such relationships exist, opening up the possibility that factors affecting these patients with poor outcome linked to widespread DNA methylation dysregulation could be identified and exploited to reduce the potential of acquired resistance to chemotherapy.

## Methods

With the biclustering problem defined in the previous section, a key innovation in the GABi approach is to utilize rule-based feature selection into the bicluster scoring process. Motivated by the fact that for many biclustering applications it will be relatively straightforward to deduce a set of the available features that provide a good representation of the specified bicluster model, given a selected subset of samples, this step eliminates the need to evaluate a potentially vast number of mostly suboptimal combinations of features for which the pattern might apply. The goal of feature selection is then:

(8) $$\begin{array}{@{}rcl@{}} \text{Given some}\,\,\,\, f(I,J), && \end{array} $$

(9) $$\begin{array}{@{}rcl@{}} \text{For any} \,\,\,J &\subseteq& Y, \end{array} $$

(10) $$\begin{array}{@{}rcl@{}} \text{find}\;I^{*} &=& {argmax}_{I \subseteq \wp(X)}\left(\,f(I,J)\right) \end{array} $$

Motivation for adopting this approach comes from the fact that in many applications it would always be desired to identify the maximal subset of the features for which that bicluster pattern holds across the given subset of samples. Then, provided the maximal subset of appropriate features could be identified for any subset of the samples represented in the data, the ‘row dimension’ of the bicluster search could be disregarded. If we define a scoring function *g*(*J*) in terms of only the columns of a bicluster, assuming selection of the optimal (or at least a near-optimal) subset of rows, then we redefine the biclustering problem:

(11) $$\begin{array}{@{}rcl@{}} {}\text{Define}\,\,\, g(\,J) &=& \max\limits_{I \subseteq \wp(X)}\left(\,f(I,J)\right) \end{array} $$

(12) $$\begin{array}{@{}rcl@{}} {}\text{Find ordering} \,\,\,\boldsymbol\tau &=& \{ \tau_{1},...,\tau_{2^{m}} \}, \end{array} $$

(13) $$\begin{array}{@{}rcl@{}} {}\text{such that}\,\,\, g(\,J_{\tau_{k}}) &\geq& g\left(J_{\tau_{k+1}}\right). \forall k \in \{ 1,...,2^{m}-1 \} \end{array} $$

Given a rule that finds the appropriate *I*^∗^ for any given subset of columns *J* from the dataset, so that *g*(*J*) can be evaluated directly and efficiently, the biclustering problem given in Equations 12–13 requires exploration of a far smaller search space than the equivalent definition from the previous section. As there will still be 2^*m*^ possible biclusters to explore, heuristic techniques may still be required for approximating ***τ*** for datasets with large *m*. Genetic Algorithms form a class of heuristic search techniques for exploring complex solution spaces, such as finding the best scoring bicluster from the set of all possible biclusters in a dataset. For a thorough description of Genetic Algorithms (GAs) see [29], but for the purposes of this paper it is sufficient to know that a GA requires a representation of any possible solution as a bit string (termed a ‘chromosome’), and a ‘fitness function’ which evaluates each solution and gives a quantitative estimate of their relative desirability. Assuming rule-based selection of the optimal feature set for some subset of samples and a definition of the desired bicluster structure, a natural GA representation of a bicluster is a bit string of length the number of columns in the data matrix. This bit string would take a value of 1 when the sample is included in the bicluster and a value of 0 otherwise. As good biclusters are likely to be composed of smaller biclusters with similar patterns across different subsets of the large bicluster’s samples, it would be likely that the GA mechanism of exploring the solution space through recombination of small, good solutions would prove to be successful. In this way, the nature of the biclustering problem presented here is similar to the ideal GA problems discussed in [23] and [29]. Furthermore, the fact that addition of a new column to an existing, good bicluster can result in a far inferior (or non-existent) pattern of consistency across the new bicluster means that local search heuristics are unlikely to perform so well due to the greater chance of adding a column that doesn’t fit the bicluster pattern (and therefore results in an inferior bicluster) than one that fits the bicluster pattern and results in a better bicluster.

A second crucial innovation of the GABi approach is the modular implementation within an R package, enabling customization of the fitness function. The fitness function is the mechanism by which desirability of solutions is encoded into the GA, and incorporates rule-based feature selection. Through custom specification of this component of the algorithm, any bicluster models can be implemented and evaluated within the GABi framework. This means that the GABi framework can be used to perform subspace pattern mining for a whole range of tasks, offering insight into a tremendous range of systems. Defining what makes a good solution is clearly a critical part of the biclustering process, whether explicit as in a GA or otherwise, and should be given due attention. The general GABi framework is defined in Algorithm 1, followed by description of a number of example fitness functions and their corresponding feature selection rules. In Algorithm 1, GetFitness corresponds to *g*(*J*) in Equation 11. An example of convergence criteria that can be used include setting a maximum number of iterations through the main loop of the algorithm (sometimes termed ‘generations’ of the GA), and exiting the loop if the fitness of the best solution in the population has not improved over a specified number of generations.

**Figure 1:**
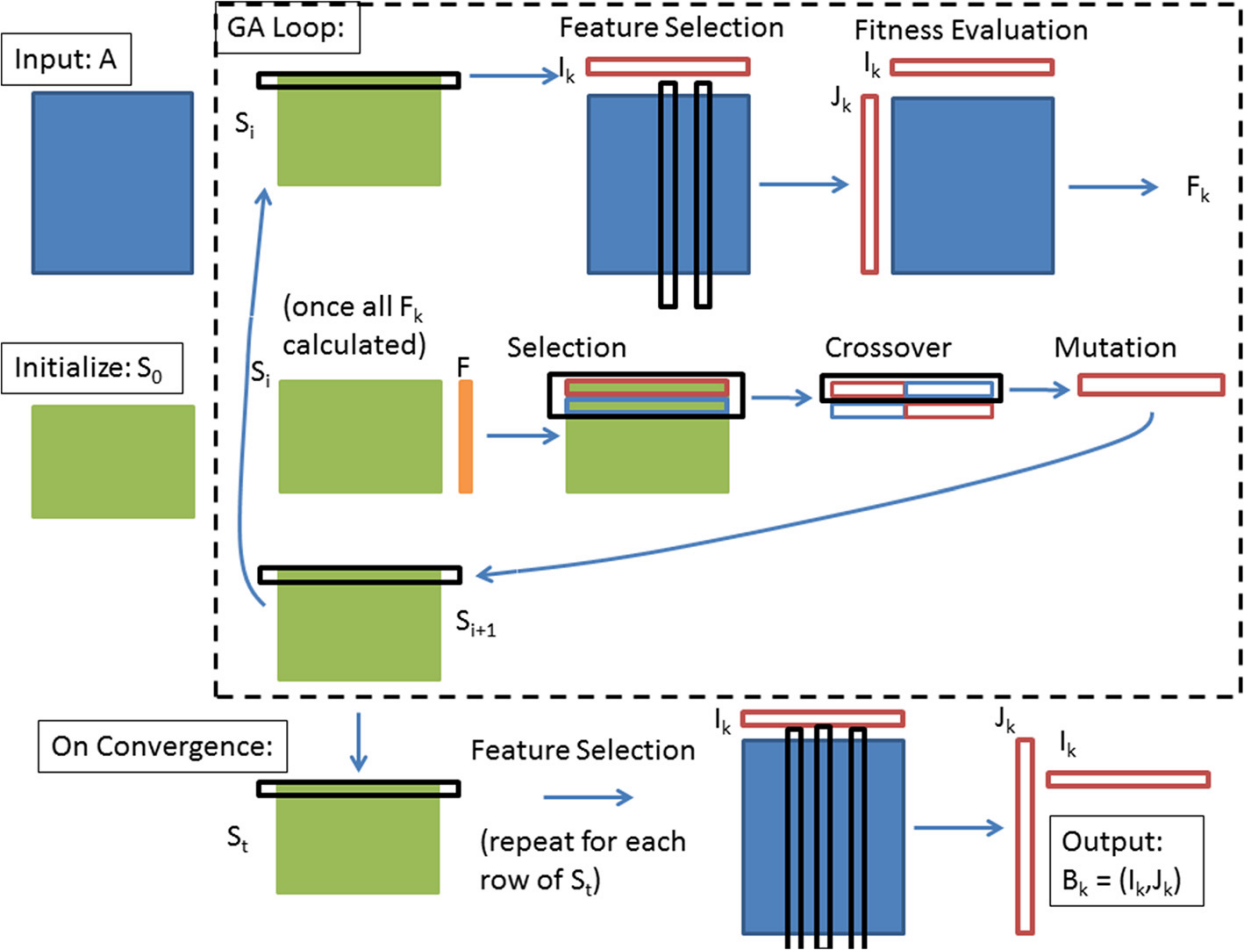
**Overview of the GABi process.** A diagram illustrating the GABi process. Starting with an input data matrix *A*, a population of candidate solutions *S* is initialized to *S*_0_. *S*, which encodes the bicluster columns *I*_*k*_ for each candidate solution *k*, is iteratively updated through the GA loop. At each step of the GA loop, each solution is evaluated in turn: rule-based feature selection is applied to identify *J*_*k*_ given *I*_*k*_ and *A* (and potentially external information), then the fitness score for the solution (*F*_*k*_) is calculated based on the function *f*(*I*_*k*_,*J*_*k*_). Based on the fitness scores *F*, candidate solutions are selected so that fitter solutions are more represented in the next iteration. Solutions are combined through crossover operations, and finally randomly ‘mutated’ before re-entering the solution population *S* as it goes through the GA loop again. When convergence criteria are met, each candidate solution’s *I*_*k*_ are extracted and the corresponding *J*_*k*_ identified through rule-based feature selection. The (*I*,*J*) sets encode the biclusters that are the output of the algorithm.



To summarize Algorithm 1, Figure 1 illustrates the overall GABi process for identifying biclusters in a data matrix *A*.

### Parameter setting

The GA function optimization framework involves setting the values of some parameters, which govern the architecture of the search space exploration. The book of [29] provides a comprehensive discussion of the impacts of these parameters on the process, but a brief summary is provided here. Essentially, the goal of the GA is to find solutions optimizing the given fitness function. Therefore, these core GA parameters will only affect how well the fitness space is explored, and will not affect the assessment of desirability of any solutions. Typically, the GA will work effectively with a range of parameter settings, with most offering a trade-off with increasing chance of discovering optimal solutions (i.e. the best biclusters) involving increasing computation time.

*PopulationSize*: this sets the number of candidate solutions to keep in the GA population. A higher number will result in a greater chance of good solutions being generated through the GA process from random initialization. We find that for most tasks, a population size of 128 is sufficient.

*Ngens*: this sets the maximum number of iterations for which the GA will run, if convergence criteria are not met before. Just a practical limit, setting this parameter will depend on how long each iteration takes (which depends on the PopulationSize and the complexity of the fitness function), the computational resources available and the length of time for which the user is prepared to wait for the algorithm to return its output. In practice, 100–200 generations is typically sufficient to reach some sort of convergence.

*SubpopSize*: the GA population may be split into ‘isolated’ sub-populations. This parameter specifies the number of such subpopulations. Splitting the overall population into these subpopulations reduces the chances of locally-optimal solutions saturating the population and reducing the algorithms efficiency at exploring complex fitness landscapes (cite). While using only 1 subpopulation is perfectly acceptable, we find that it can benefit peformance to use up to 4 subpopulations (each with at least 32 individual candidate solutions).

*Crossover rate*: this is the proportion of candidate solutions at each GA iteration that will exchange information (I.e. Swap substrings of their bit patterns) with one another. Setting it too low will slow down the progress of the GA, such that a value of zero will result in a parallelized version of the ‘random mutation hill climber’ (although this in itself can be a reasonable function optimizer). Typically it will be in the range 0.5–1.

*Mutation rate*: this is the probability that each bit in each candidate solution will be flipped at each generation. Setting this value too high will mean good candidate solutions are frequently disrupted too much to contribute to subsequent generations, and thus the algorithm doesn’t really progress. Setting it too low will slow the rate at which the GA explores the given fitness landscape. Typically, a good guideline value is approximately 1/solutionLength (that is, 1/n, where n is the number of samples in the dataset).

*MaxLoop*: this parameter enables some flexibility in the way the GABi framework is used. While the core GA is inherently parallel, sometimes a combination of dataset and fitness function supplied will result in one high-scoring solution dominating the algorithms population(s). When the MaxLoop parameter is set to a value greater than 1, upon convergence of the GA, GABI will save the output solutions, add them to a ‘tabu list’ that will have zero fitness in subsequent runs, and repeat the entire GA process. This will continue MaxLoop times, enabling more diverse solutions to be discovered. Its effective use is often not necessary, and depends on appropriate handling of the ‘tabu list’ in the fitness function. However, if you wish to discover a broader range of biclusters in your dataset that fit the supplied pattern, you can try setting this parameter.

An important practical point to note is that the fitness function will be called many times throughout the algorithm’s progress, and so the desirability of a solution should be expressed in such a way that the value may be calculated very quickly. The implementation of a bicluster search algorithm able to use different bicluster definitions and desirability models is the primary motivation for this work, and so the fitness function of the GABi algorithm is intentionally left unspecified with the following constraints: it must identify the maximal set of features fitting the desired bicluster definition across a specified set of samples, and it must calculate a numerical value proportional to the desirability of the bicluster formed by the specified samples and the identified features. This function may operate on any type of data matrix, with values normalized or discretized in any fashion. We now define number of different bicluster models that were utilized for the work presented in this paper (for R code, see Additional file 1: Supplementary Information S1). Definitions neccessarily include a submatrix-based bicluster scoring function *f*(*I*,*J*), a feature selection rule *δ*_*i*_ (which represents the means of finding *I*^∗^ in Equation 10, and a column-wise bicluster scoring function *g*(*J*) that incorporates *δ*_*i*_. One final point regarding the handling of customized fitness functions in GABi is that it is possible to involve any number of additional parameters, or even additional datasets, in the fitness evaluation. As demonstrated with the example in Section ‘Associating dysregulation of DNA methylation with ovarian cancer patient prognosis’, this means that it is possible to use GABi for intelligent integration of multiple data sources across heterogeneous subjects - providing a means to address one of the most critical bottlenecks in utilization of data to guide biological research and discovery.

### Block bicluster model

In a binary data matrix, an obvious definition of a bicluster is a region of local density. That is, some submatrix in which all (or most of) the values are 1. Under the principle that the larger the pattern, the better, the relative desirability of a basic bicluster is simply the product of the number of rows and the number of columns. The selection rule requires specification of a parameter *θ*, the minimum proportion of the bicluster columns with 1s for each row *i*. This allows some flexibility when applied to noisy data.

(14) $${} {\small{\begin{aligned} \text{Define}\; f^{block}(I,J) \,=\, |I||J|, \text{where} |J| \text{denotes the cardinality of set}\, J \end{aligned}}}  $$

(15) $${\kern26pt} {\small{\begin{aligned} \delta^{block}_{i} =\left\{ \begin{array}{l} 1, \; \text{if}\, \displaystyle \frac{1}{|J|} \sum\limits_{j \in J} a_{ij} \geq \theta\\ 0, \; \text{otherwise} \end{array} \right. \end{aligned}}}  $$

(16) $${\kern16pt} {\small{\begin{aligned} g^{block}(J) = |J|\displaystyle \sum\limits_{x \in X} \delta^{block}_{x} \end{aligned}}}  $$

While intended for binary matrices, this model can also apply to non-binary numeric matrices, either directly or by initially binarizing the matrix (e.g. setting all values above a specified quantile or value to be 1s, and all other values 0s). In relation to the 4 bicluster types defined in the previous section, the block bicluster model is clearly designed to discover biclusters of type 1. With row-wise or column-wise binarization of the dataset, this could also be used to discover biclusters of type 2.

### Correlation bicluster model

The biclustering version of correlation is a subset of the columns of a data matrix, over which a subset of the rows all show significant pairwise correlation. We define an effective scoring function for identifying correlation biclusters, involving identification of a ‘seed row’ *i*^∗^, then taking the sum of the negative logarithms of the p-values of each selected row’s correlation (across the selected columns) with the seed row, and multiplying this by the number of columns in the bicluster. In more formal terms, based on significance estimates of the Spearman correlation coefficient, with *Φ* denoting the standard Normal cumulative distribution function, *abs*(.) representing the absolute value of its argument, and *α* representing a maximum p-value for selecting only features with significant correlations to the seed row:

(17) $${} {\small{\begin{aligned} \text{Say}\; c_{ik} = \displaystyle \frac{\displaystyle \sum\limits_{l=1}^{|J|}(a_{{ij}_{l}}-\overline{A}_{iJ})(a_{{ik}_{l}}-\overline{A}_{iJ})}{\sqrt{\displaystyle \sum\limits_{l=1}^{|J|}(a_{{ij}_{l}}-\overline{A}_{iJ})^{2} \displaystyle \sum\limits_{l=1}^{|J|}(a_{{ik}_{l}}-\overline{A}_{iJ})^{2}}},\\ \end{aligned}}}  $$

(18) $${} {\small{\begin{aligned} \text{where}\,\,\, \overline{A}_{iJ} = \displaystyle \frac{1}{|J|} \sum\limits_{j \in J} a_{ij}. \end{aligned}}}  $$

(19) $${} {\small{\begin{aligned} \text{Then define}\; f^{cor}(I,J) = -|J|\!\displaystyle \sum\limits_{i \in I}\!\log\! \left(\! 2 \Phi \!\left(\!\!\sqrt{\!\displaystyle\frac{|J|\,-\,3}{1.06}}arctanh(c_{ii^{*}}\!)\!\right)\!\! \right)\!, \end{aligned}}}  $$

(20) $${} {\small{\begin{aligned} \text{Where}\; i^{*} = {argmax}_{x \in X}\displaystyle\!\left(\!abs \!\left(\! \displaystyle \frac{\sum_{l=1}^{|J|}\left(a_{{ij}_{l}}\,-\,\overline{A}_{iJ}\right)\left(b_{l} \,-\, \overline{b}\right)}{\sqrt{\displaystyle \!\sum\limits_{l=1}^{|J|}\!\left(a_{{ij}_{l}}\,-\,\overline{A}_{iJ}\right)^{2}\sum\limits_{l=1}^{|J|}\!\left(\!b_{l} \,-\, \overline{b}\right)^{2}}}\! \right)\!\! \right)\!, \end{aligned}}}  $$

(21) $${} {\small{\begin{aligned} \text{where} \;\; \mathbf{b} &= \left\{ b_{1},\ldots,b_{|J|} \right\}\;\text{is the rotation vector for the first}\\ &\quad\text{principal component of} \,\,\, A_{XJ}.\\[-15pt] \end{aligned}}}  $$

(22) $${} {\small{\begin{aligned} \delta^{cor}_{i} =\left\{ \begin{array}{l} 1, \,\,\,\text{if} \,\,\,\displaystyle 2 \Phi \left(\sqrt{\displaystyle\frac{|J|-3}{1.06}}arctanh(c_{ii^{*}})\right) < \alpha \\ 0, \,\,\, \text{otherwise} \end{array} \right. \end{aligned}}}  $$

(23) $${} {\small{\begin{aligned} g^{cor}(J) = -|J|\displaystyle \sum\limits_{x \in X}\log \left(\! 2 \Phi \!\left(\!\sqrt{\displaystyle\frac{|J|\,-\,3}{1.06}}arctanh(c_{xi^{*}})\right)\! \right)\delta^{cor}_{x} \end{aligned}}}  $$

The correlation bicluster model is clearly designed to discover biclusters of type 3, which includes type 2 biclusters with constant columns as these will necessarily involve a shared pattern preserved across the rows. It should also prove moderately effective in discovering biclusters of type 4.

### High-variance bicluster model

One possible scenario of interest for biclustering is the identification of a submatrix with particularly high variance when compared to the rest of the matrix. To discover these biclusters, a scoring function was created that first selects rows for which the variance across the selected columns is significantly greater than the variance across all the unselected columns. The score is then the sum of the negative logarithms of the p-values of each selected row’s difference in variance between the bicluster columns and the rest of the data matrix, multiplied by the number of columns in the bicluster. More formally, with $F^{\chi ^{2}}(x;k)$ denoting the cumulative distribution function of the chi-squared distribution of *x* with *k* degrees of freedom, $\sigma ^{2}_{i_{J}}$ denoting the variance of row *i* of the data across columns *J*, and *α* again representing a maximum p-value for selection of features with a difference in variance that is sufficiently significant:

(24) $${} \text{Define}\,\,\, f^{var}(I,J) = -|J|\displaystyle \sum\limits_{i \in I}\log(p_{i}),  $$

(25) $${} \text{where}\,\,\, p_{i} = F^{\chi^{2}}\left(\displaystyle \frac{\sigma^{2}_{iJ}}{\sigma^{2}_{iJ'}}; |J|-1 \right),  $$

(26) $${} \text{and}\,\,\, J^{\prime} = Y \setminus J  $$

(27) $$ \quad\;\,= \{ y | y \notin J\}.\forall y \in Y  $$

(28) $${} \delta^{var}_{i} =\left\{ \begin{array}{l} 1, \,\,\, \text{if}\,\,\, p_{i} < \alpha \\ 0, \,\,\, \text{otherwise} \end{array} \right.  $$

(29) $${} g^{var}(J) = -|J|\displaystyle \sum\limits_{x \in X}\log(p_{i})\delta^{var}_{x}  $$

A simple example designed to give an indication of the flexibility of the GABI framework, this model does not fit any of the traditional bicluster types 1-4. It is related to the ‘variance biclusters’ of HSSVD [21], which represented the first example of biclusters being defined based on the variances of the values within the bicluster.

### Dysregulation-vs-outcome bicluster

This represents a very specific adaptation of the correlation bicluster, in which rows are only included in the bicluster if there is some set of columns (outside the bicluster) across which they are largely uncorrelated with one another. Furthermore, the score of each bicluster is multiplied by the negative logarithm of the p-value arising from the logrank test of the difference in progression-free survival time between the patients corresponding to columns of the bicluster and the patients corresponding to the columns across which the selected rows are uncorrelated. Formally, using *O*_*Jt*_ to denote the number of observed events (deaths) in population *J* at time *t*, and *N*_*Jt*_ to denote the number of samples (patients) in population *J* that have neither had an event nor been censored (removed from the study or lost to follow-up) at time *t*:

(30) $${} \text{Define}\,\,\, f^{surv}(I,J) = \max\left(g^{cor}(J), g^{block}(J) \right)h(I,J)  $$

(31) $${\kern8pt} (I,J) = -log\left(2 \Phi \left(\displaystyle \frac{\sum_{t=1}^{T}(O_{Jt}-E_{Jt})}{\sqrt{\sum_{t=1}^{T}V_{t}}} \right) \right),  $$

(32) $${} \text{where}\,\,\, E_{Jt} = \displaystyle\frac{O_{t}}{N_{t}}N_{Jt}  $$

(33) $${} \text{and}\,\,\, V_{t} = \displaystyle \frac{O_{t}(\frac{N_{Jt}}{N_{t}})(1-\frac{N_{Jt}}{N_{t}})(N_{t}-O_{t})}{N_{t}-1},  $$

(34) $${\kern5pt} O_{t} = O_{Jt} + O_{J^{\prime}t},  $$

(35) $${\kern5pt} N_{t} = N_{Jt} + N_{J^{\prime}t},  $$

(36) $${} \text{where}\,\,\, J^{\prime} = \{ y_{r_{1}},...,y_{r_{l}} \},  $$

(37) $${\kern18pt} l = \min(|J|,|Y|-|J|),  $$

(38) $${} \text{and}\,\,\, \mathbf{r} = \{ r_{1},...,r_{|Y|}\}, \text{is a ranking of Y such that:}  $$

(39) $${\kern10pt} S^{I}(y_{r_{k}}) \leq S^{I}(y_{r_{k+1}})  $$

(40) $${} {\small{\begin{aligned} \text{where}\,\,\, S^{I}(y_{r_{k}}) = abs\!\left(\! \begin{array}{l} \!\left(\displaystyle\! \frac{\displaystyle \sum\limits_{i \in I}\!\left(a_{{iy}_{r_{k}}}\,-\,\overline{A}_{{Iy}_{r_{k}}}\!\right)\left(a_{{ij}_{1}}\,-\,\overline{A}_{{Ij}_{1}}\right)}{\sqrt{\displaystyle \sum\limits_{i \in I}\!\left(a_{{iy}_{r_{k}}}\!\,-\,\overline{A}_{{Iy}_{r_{k}}}\right)^{2} \displaystyle \sum\limits_{i \in I}\!\left(a_{{ij}_{1}}\,-\,\overline{A}_{{Ij}_{1}}\right)^{2}}}\!\right)\! \end{array}\!\!\! \right) \end{aligned}}}  $$

(41) $${\kern5pt} \delta^{surv}_{i} =\left\{ \begin{array}{l} \delta^{block}_{i},\,\,\, \text{if}\; g^{block}(J) > g^{cor}(J) \\ \delta^{cor}_{i}, \,\,\, \text{otherwise} \end{array} \right.  $$

(42) $${} g^{surv}(J) = \left\{ \begin{array}{l} g^{block}(J)h(I^{\prime},J), \,\,\, \text{if}\; g^{block}(J) > g^{cor}(J) \\ g^{cor}(J)h(I^{\prime{\prime}},J),\; \text{otherwise} \end{array} \right.  $$

(43) $${} \text{where}\,\,\, I^{\prime} = \{ x_{i} | \delta^{block}_{i}=1 \}.\forall x_{i} \in X  $$

(44) $${} \text{and}\;\, I^{\prime\prime} = \{ x_{i} | \delta^{cor}_{i}=1 \}.\forall x_{i} \in X  $$

In Equation 36, **r** gives the ranking of all columns in terms of increasing absolute value of correlation (across selected features) with the first column contained in the bicluster (that is, *j*_1_).

Another example that illustrates the flexibility of the GABI framework, the specific (and somewhat esoteric) use of the two input data sources involved in this model means that it is not related to any of the traditional bicluster types 1-4. No existing biclustering method displays the capacity to identify biclusters fitting such application-specific models as this. The main advance of the GABI framework is the ability to address such biclustering problems.

## Results

The main aim of introducing the GABi framework to the field is to provide a fully flexible bicluster search tool that enables discovery of submatrices reflecting good representations of any desired pattern from within a data matrix. GABi is not a tool intended to perform any one particular biclustering task better than the state-of-the-art among existing approaches, rather it is intended to broaden the range of data mining tasks to which biclustering can be applied. Therefore, it would be somewhat missing the point of this work to carry out a straightforward comparison to the existing state-of-the-art methods using standard benchmarking datasets (such as those of [19,20]) that reflect the tasks they were designed to perform. Instead, the strategy taken here to demonstrate the success of the GABi framework in achieving its goal is to apply GABi with a number of different bicluster models to datasets that reflect the scenarios for which they were designed. The framework is clearly successful if the bicluster model matched to the appropriate task yields significantly better results than using a bicluster model designed to discover a different type of pattern. Three scenarios using artificial datasets are presented, and one using genome-wide DNA methylation profiles from ovarian cancer samples.

### Bicluster discovery in synthetic data

Artificial datasets provide the opportunity to implant patterns of defined structure into known locations within data matrices. The ability of an algorithm to discover the intended patterns (i.e. find the locations into which the simulated patterns were implanted) can be readily evaluated by calculating overlap between the solutions returned by the algorithm and the implanted patterns. Overlap between two biclusters can be quantitatively assessed by calculating an F-measure. The F-measure incorporates both precision and recall, as shown in the following equation giving the formula for the F-measure for overlap of two sets A and B (in which ∣*A*∣ denotes the number of elements in set *A*, and *A*∩*B* denotes the intersection of sets A and B):

(45) $$ F = 2*\frac{\frac{\mid A \cap B\mid}{\mid A\mid}*\frac{\mid A \cap B\mid}{\mid B\mid}}{\frac{\mid A \cap B\mid}{\mid A\mid}+\frac{\mid A \cap B\mid}{\mid B\mid}}  $$

Using this framework, A and B represent the features of the recovered and implanted biclusters, respectively. To get an overall measure of the overlap between a recovered bicluster and an implanted bicluster, the F-measure for the features was multiplied by the F-measure for the samples. As each implanted bicluster could be represented by any one of the recovered bicluster solutions (or none at all), the best F-measure for each implanted bicluster with any of the bicluster solutions recovered from the respective dataset was taken as a dataset-oriented measure of the success of a set of recovered biclusters.

For each of the first three bicluster models defined in the previous section of this paper (that is, the ‘block bicluster model’, ‘correlation bicluster model’ and ‘high-variance bicluster model’), a structure of implanted data was created to reflect the specific type of pattern the model was intended to discover. The general strategy for artificial dataset creation is given in Algorithm 2, with individual data structures defined as follows:



#### Block bicluster model

For the block bicluster model, draw a single offset *b* from *N*(0,1). Add this value *b* across submatrix defined by (*I*,*J*).

#### Correlation bicluster model

For the correlation bicluster model, create a vector of offsets **b**={*b*_1_,...,*b*_*q*_} with all *b*_*j*_ drawn randomly from *N*(0,1). For each *j*∈*J*, add the value *b*_*j*_ to column *j*, across all bicluster rows *I*.

#### High-variance bicluster model

For the high-variance bicluster model, create a *p*×*q* matrix of offsets *B*=[*b*_*ij*_], with all *b*_*ij*_ drawn randomly from *N*(0,1). For each row *i* in the bicluster, for each column *j* in the bicluster, add the corresponding *b*_*ij*_.

#### Artificial dataset results

A set of 40 synthetic datasets were constructed for each of these bicluster models, using the strategy outlined earlier in this section. For all datasets, *n*=100, *m*=20, *p* was randomly drawn from {1,...,100}, *q* was randomly drawn from {2,…,18} (in order to ensure there were at least 2 columns in the bicluster and at least 2 columns out of the bicluster), and *ζ* was randomly drawn from *U*(0,1).

In addition to using GABi with the three specified bicluster models, a number of widely used biclustering algorithms were applied to the same artificial data analysis tasks: ISA [15], Cheng & Church [10], Plaid [11] and Bimax [19]. This provides an indication of the baseline performance that would be expected of any bicluster algorithm when applied to the given task, regardless of the bicluster model. It is therefore expected that if the GABi framework functions effectively, the implanted data structure reflects the ideal bicluster structure according to one of the bicluster models, and the noise added to the artificial datasets does not obscure the signal implanted into the data, then GABi with the appropriate bicluster model will achieve higher F-scores than any of the alternative methods (including GABi with an inappropriate bicluster model).

**Figure 2:**
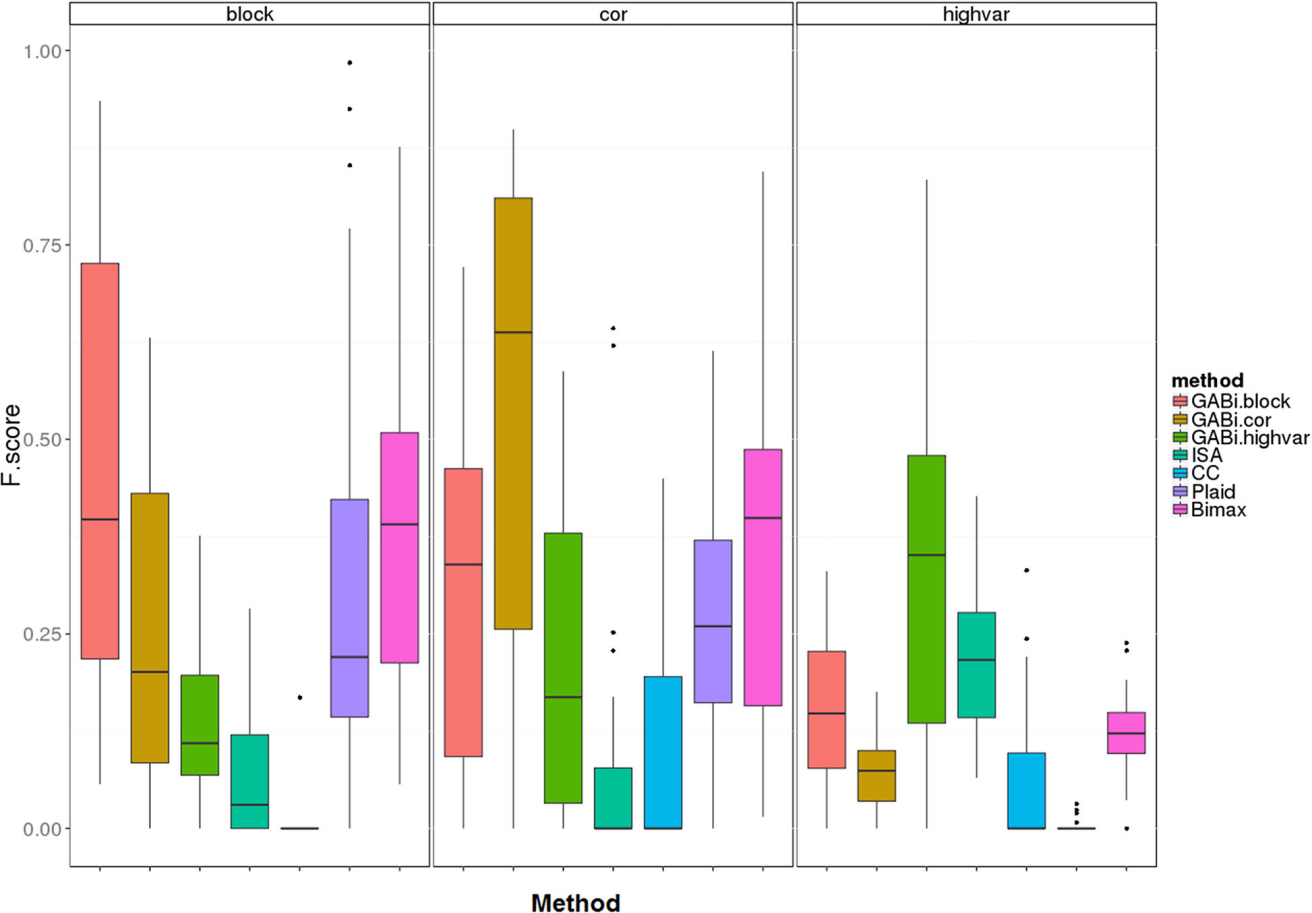
**Structure-dependent biclustering algorithm recovery scores.** Distribution of per-dataset best overlap scores for each biclustering algorithm. Scores for GABi framework with the ‘naïve’ bicluster model are shown in red, scores for GABi framework with the ‘correlation-based’ bicluster model are shown in green, and scores for ISA are shown in blue. The panel on the left shows the results for datasets with ‘Block Structure’ and the panel on the right shows the results for datasets with ‘Correlation Structure’.

The distributions of each dataset’s best-overlap scores for each biclustering algorithm’s corresponding set of solutions are shown in Figure 2. It is clear from inspection of the distributions of implanted bicluster recovery scores that the GABi framework recovered implanted solutions with a ‘Block Structure’ better using the block bicluster model than either the correlation model or the high-variance model, that the GABi framework recovered implanted solutions with a ‘Correlation Structure’ better using the correlation bicluster model than either the block bicluster model or the high-variance model, and that the GABi framework recovered implanted solutions with a ‘High-variance Structure’ better using the high-variance bicluster model than either the block bicluster model or the correlation model. Additionally, when using the appropriate bicluster model for a given dataset, the GABi framework recovered the implanted solutions better than any of the other methods used for comparison. These comparisons were all statistically significant (at *p*<0.01) according to Wilcoxon signed rank test, with the exception of a marginally-significant comparison to Bimax on recovering implanted solutions with a ‘Block Structure’ (*p*=0.1). This is not particularly surprising, given that the other methods were not specifically designed for identifing biclusters of the implanted structures, although the ‘Block Structure’ resembles the bicluster model of Bimax and is a special case of the model of the Plaid method. Crucially, these evaluations shows that the GABi framework can be adapted to different data analysis tasks, and successfully discovers biclusters with the intended structure. This adaptability is shown to be specific to the extent that performance of methods using bicluster models that don’t reflect the implanted data structure is never as good.

### Associating dysregulation of DNA methylation with ovarian cancer patient prognosis

A key aspect of the GABi framework is that it can incorporate multiple sources of information to guide bicluster discovery. This could involve prior beliefs about which features may be the most relevant to an investigation, or could involve additional data sources. In the following application, patient outcome data was incorporated into the bicluster evaluation process to guide bicluster discovery towards clinically relevant observations.

DNA methylation profiles of primary high grade serous ovarian tumour samples from Illumina HumanMethylation27k BeadChips, along with clinical data for patients from whom the samples were taken, were obtained from The Cancer Genome Atlas (TCGA) data portal [30]. Level 3 methylation data was used, consisting of probe level *β*-values. Loss of stability of DNA methylation at certain genomic loci has been shown to be a feature of cancers [21,28]. However, it has not yet been established if there are regions for which the DNA methylation is tightly controlled across some tumours and not others. Nor has it been established if the loss of epigenetic stability in any such regions is associated with a more aggressive tumour phenotype and worse patient outcome.

The ‘dysregulation-vs-outcome bicluster model’ (defined in the ‘Methods’ section of this paper) was developed to offer insight into these questions. It involves identifying rows that show correlated profiles within the bicluster, and for which there is some set of columns outside the bicluster across which they are largely uncorrelated with one another. Furthermore, the score of each bicluster is multiplied by the negative logarithm of the p-value arising from the logrank test of the difference in progression-free survival time between the patients corresponding to columns of the bicluster and the patients corresponding to the columns across which the selected rows are uncorrelated.

Using this model for bicluster search, GABi identified a set of 21 samples that showed a high level of correlation of DNA methylation across 14,382 CpG sites across the genome (Illumina HumanMethylation CpG probe identifiers are listed in Additional file 2: Table S2). A ‘control’ set was identified, consisting of the 21 samples with lowest correlation to the bicluster samples, across the features selected in the bicluster model.

**Figure 3:**
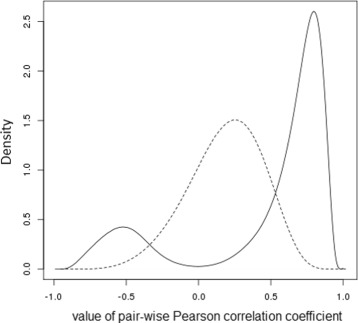
**Pair-wise correlation of CpG methylation levels in subgroups of ovarian cancer.** Kernel density estimation plots of the complete set of Pearson correlation coefficients between each pair of CpG sites selected in the DNA methylation dysregulation bicluster. Distribution of correlation coefficients across the subset of tumours selected in the bicluster shown with solid line, corresponding distribution of correlation coefficients across the subset of ‘control set’ of tumours with dysregulation of this DNA methylation.

**Figure 4:**
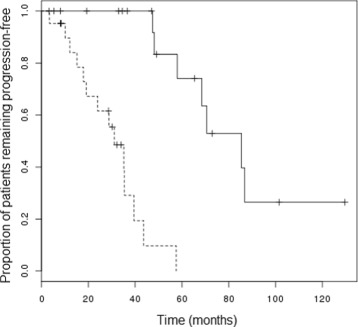
**Progression-free survival of subgroups of ovarian cancer, identified through biclustering.** Plot of Kaplan-Meier estimates of progression-free survival for subset of tumours identified through bicluster analysis as having coherently regulated levels of DNA methylation of 14,382 CpG sites (solid line), compared with the subset representing a ‘control set’ of tumours with dysregulation of this DNA methylation.

**Figure 5:**
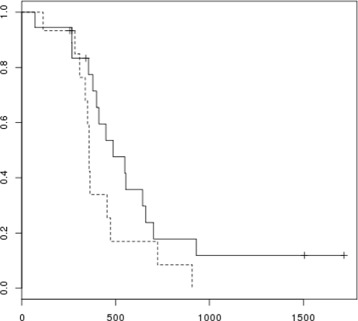
**Progression-free survival of ovarian cancer patients from validation cohort, classified according to correlation of DNA methylation profiles.** Plot of Kaplan-Meier estimates of progression-free survival for two subsets of tumours from the validation cohort: one (solid line) showing greatest correlation of DNA methylation across 14,382 CpG sites to the profile identified from the bicluster in the TCGA dataset, compared with the subset showing least correlation to this DNA methylation profile.

Two observations regarding this set of samples are of particular note. Firstly, the correlation coefficients between the methylation levels of all pairs of selected CpG sites shows a distinctly skewed distribution across the bicluster samples (Figure 3, solid line). Compare this to the corresponding distribution of correlation coefficients calculated between the same CpG sites, but across the selected ‘control set’ of samples (Figure 3, dashed line). It is clear that the DNA methylation levels at these 14,382 CpG sites are indeed highly correlated with one another across the selected bicluster samples, but not particularly correlated at all across the control set of samples. Secondly, the bicluster samples have markedly better prognosis than the control samples (Kaplan-Meier plot shown in Figure 4, logrank test *p*=1∗10^−5^). In fact, the median time to relapse or death for the patients whose tumour samples were in the bicluster set, at 85.4 months, was more than twice as long as that of the patients whose tumour samples were in the control set (32 months). In an attempt to aseess the generality of this observation, an additional DNA methylation dataset was obtained from profiling 78 primary high grade serous ovarian tumours with the Illumina HumanMethylation27k Bead Chip platform. Samples were ranked according to the absolute value of the Pearson correlation coefficient of their DNA methylation *β*-values with those of the first sample from the bicluster in the TCGA dataset, across the 14,382 CpG sites defined by the bicluster. No samples in this independent dataset were as uncorrelated with the bicluster sample as those of the ‘control set’ of samples from the TCGA dataset, and so it is difficult to draw any conclusions from this, other than the fact that such extreme epigenetic dysregulation as was identified a subset of samples from the TCGA dataset may represent a rare phenotype. However, it was interesting to note that outcome (in terms of time to death and time to relapse) was significantly better for the 20 patients whose tumours were most correlated with the bicluster sample, against the 20 patients whose tumours were least correlated with the bicluster sample (logrank test for overall survival *p*=0.1, for progression-free survival *p*=0.15, Kaplan-Meier plot for progression-free survival shown in Figure 5). DNA methylation profiles and outcome data for these patients are provided in Additional file 3: Table S3.

These results suggest that, by incorporating progression-free survival data into the bicluster model used by GABi, it has been possible to demonstrate that systematic dysregulation of DNA methylation in ovarian cancer can be associated with a marked worsening of the patients’ prognosis.

## Discussion

The GABi framework introduces a paradigm for discovering good examples of potentially *any* desired pattern across any subset of a dataset. The freely available R package enables heuristic problem solving according to custom bicluster models, embedding rule-based feature selection within a genetic algorithm for bicluster discovery. In essence, this means that rather than having to create a whole new search algorithm every time you want to discover a new type of pattern in subsets of some dataset(s), you just need to define a feature selection rule and a scoring scheme. The principal advance of this work, and in fact its underlying motivation, is that it has the potential to broaden the range of suitable applications for biclustering methods, through application-specific definitions of bicluster models. The evaluation of the GABi framework in this paper shows that it can be adapted to perform different tasks, including those for which no other biclustering algorithm could be used.

This advance is especially exciting in the context of the heterogeneity that is becoming apparent in many large-scale biological datasets, as the concept of identifying patterns within *a priori* unknown subsets of a data matrix is particularly well suited to application to such data. Furthermore, the flexible bicluster definition enables intelligent integrated analysis of multiple heterogeneous datasets, as demonstrated by the application to find links between patterns of DNA methylation and patient outcomes in ovarian cancer. The potential for biclustering to be used as a means of integrating diverse collections of datasets was first illustrated in [31], but again the flexible design of the GABi framework opens up a vast array of potential applications of biclustering to data mining across multiple collections of data.

One point worth noting regarding this approach is that the application of GABi to large collections of data is dependent on having a bicluster evaluation model that is sufficiently fast to compute. One potential avenue for future work is the development of a parallel implementation of GABi. The population-based architecture of the GA inherently lends itself to parallel computing, as the fitness evaluations at each iteration of the algorithm can be performed independently of one another. Such a parallel implementation could extend the feasible application of GABi with more complex evaluation functions, through the utilisation of multiple processors at each iterative step of the population evolution.

An application was presented using the GABi framework to perform bicluster analysis to identify clinically-relevant dysregulation of DNA methylation data in high-grade serous ovarian cancers. It is particularly interesting that this result links the dysregulation of DNA methylation that has previously been associated with cancer to differences in the propensity for rapid recurrence of the disease. That such widespread epigenetic dysregulation was observed in a rare subset of ovarian tumours warrants further investigation, to determine whether or not this feature is present and similarly linked with outcome in other cohorts of ovarian cancer or other malignancies. However, these results demonstrate that biclustering methods implemented through GABi represent suitable tools for integrated analysis of heterogeneous cancer datasets to discover clinically relevant patterns in molecular measurements.

## Conclusions

This work represents an extension of the biclustering problem from the manner in which it has traditionally been applied to molecular biology datasets (typically gene expression microarray data). Based on this generalized subspace pattern mining, an R package ‘GABi: a generalised framework for biclustering’ has been produced to implement a mechanism of discovering patterns according to any specified bicluster model. This package is freely available from CRAN (http://cran.r-project.org/web/packages/GABi/index.html). It has been demonstrated that GABi can successfully discover biclusters with properties defined by the particular bicluster model used. Using an application-specific model of biclustering, systematic dysregulation of DNA methylation has been shown for the first time to be linked with patient prognosis in ovarian cancer. This work lays the foundations for a wide range of problem-specific applications of the biclustering paradigm for pattern mining in biological data, with the application to ovarian cancer illustrating the potential of biclustering as a tool for analysis of heterogeneous cancer datasets.

## Availability and requirements

The GABi package is implemented in R, and is made available through CRAN (http://cran.r-project.org) under the GNU GPL license. The project home page is http://cran.r-project.org/web/packages/GABi/index.html.

This implementation is platform-independent, but requires a current installation of the **R** statistical programming environment.

## Additional files

Additional file 1
**Supplementary Information S1.** R code for fitness functions and feature selection rules implementing the bicluster models provided and used in this paper.

Additional file 2
**Table S2.** List of Illumina HumanMethylation array probe identifiers for the 14,382 CpG sites identified in the DNA methylation dysregulation bicluster.

Additional file 3
**Table S3.** Table of clinical outcomes and Illumina HumanMethylation array *β*-values for the 14,382 CpG sites identified in the DNA methylation dysregulation bicluster.
